# Integrated Analysis of Transcriptome Data Revealed AURKA and KIF20A as Critical Genes in Medulloblastoma Progression

**DOI:** 10.3389/fonc.2022.875521

**Published:** 2022-04-27

**Authors:** Bo Liang, Yan Zhou, Jiji Jiao, Lixia Xu, Yan Yan, Qiaoli Wu, Xiaoguang Tong, Hua Yan

**Affiliations:** ^1^ Clinical College of Neurology, Neurosurgery and Neurorehabilitation, Tianjin Medical University, Tianjin, China; ^2^ Department of Neurosurgery, The Fifith Affiliated Hospital of Zhengzhou University, Zhengzhou, China; ^3^ Tianjin Neurosurgical Institute, Tianjin Key Laboratory of Cerebrovascular and Neurodegenerative Diseases, Tianjin Huanhu Hospital, Tianjin, China; ^4^ Clinical Laboratory, Tianjin Huanhu Hospital, Tianjin, China; ^5^ Department of Neurosurgery, Tianjin Huanhu Hospital, Tianjin, China

**Keywords:** Medulloblastoma, Bioinformatics, Biomarker, AURKA, KIF20A

## Abstract

Medulloblastoma is the neuroepithelial tumor with the highest degree of malignancy in the central nervous system, accounting for about 8% to 10% of children’s brain tumors. It has a high degree of malignancy and is easily transmitted through cerebrospinal fluid, with a relatively poor prognosis. Although medulloblastoma has been widely studied and treated, its molecular mechanism remains unclear. To determine which gene plays a crucial role in medulloblastoma development and progression, we analyzed three microarray datasets from Gene Expression Omnibus. Gene Ontology and Kyoto Encyclopedia of Genes and Genomes were used to detect and evaluate differentially expressed genes. Protein interaction network was established, and the hub genes were determined in cytoHubba through various assessment methods, while the target genes were screened out using survival analysis. Ultimately, human medulloblastoma samples were utilized to confirm target gene expression. In conclusion, This study found that aurora kinase A (AURKA) and kinesin family member 20A (KIF20A) may be involved in the initiation and development of medulloblastoma, have a close association with prognosis, and may become a potential therapeutic target and prognostic marker of MED.

## Introduction

Medulloblastoma is an embryonal cerebellar tumor most commonly seen in children as a malignant brain tumor, occupying 8% to 10% of all pediatric brain cancers and occurring extremely infrequently in adults. Wingless (WNT), sonic hedgehog (SHH), group 3 (G3), as well as group 4 (G4) medulloblastoma are four primary molecularly and histopathologically different categories of medulloblastoma (1). Multimodal therapy, which included surgery, radiation therapy, and chemotherapy, decreased the late death’s cumulative incidence but raised the prevalence of recurrent tumors and critical, incapacitating chronic health disorders (2). Medulloblastoma research has also been on the leading edge of cancer genomics (3). According to current molecular genetic evidence, many molecular abnormalities are linked to cell transformation in medulloblastoma. HER2 was reported to be present in 86% of medulloblastomas and to be co-expressed with HER4 in 54% of MEDs. Co-expression of HER2 and HER4 results in a bad prognosis. In sporadic medulloblastoma, oncogenic mutations in the β -catenin gene boost transcription of numerous genes, including cyclin D1, C-MYC, and T-cytokine (TCF-1).Mutations that activate β -catenin may cause cell transformation in a medulloblastoma subpopulation (4). Clinical sequencing can convey valuable information to help with the categorization and diagnosis of MED, and some gene variations are linked to distinct molecular subtypes. CTNNB1 mutations, for example, are a particular manifestation of the WNT molecular subtype and have diagnostic relevance (5, 6). SHH tumors exhibit mutations in SHH pathway mediators, including SUFU, PTCH1, or SMO. Complete or partial deletion of chromosome 6 is usually employed to establish the diagnosis of the WNT molecular population. A positive FISH result on homologous chromosome 17q may assist to more firmly designate malignancies as MB owing to its relatively high specificity (7). The use of appropriate targeted therapies for patients with specific genetic mutations is one direction of research for molecular therapy of medulloblastoma, Patients with phosphatase and tensin homologous (PTEN) or mammalian target of rapamycin (mTOR) or PIK3CA-activated mutations, for example, were given PI3K or mTOR inhibitors, while patients with BRAF V600E mutations were given BRAF inhibitors. Patients with fibroblast growth factor receptor (FGFR) activation mutations or fusions were given FGFR inhibitors (8). Some studies have found that tumor immune microenvironment is influential in supporting or preventing tumor advancement. Tumor-associated macrophages/microglia (TAMs) can accelerate the development of tumor in the medulloblastoma sonic hedgehog subgroup (SHH-MB), As TAMs generally rely on the colony-stimulating factor 1 receptor (CSF1R), the inhibition of CSF1R may have curative promise in SHH-MB patients (9). The downregulation of miR-204 was associated with low survival rates in the G4 medulloblastom, while, in medulloblastoma cells, tumor suppression of miR-204 and miR-30a is triggered by inhibition of autophagy. Autophagy inhibitors can effectively reduce cranial spinal radiation dose, thus significantly reducing treatment-related side effects, which has potential in the treatment of medulloblastoma (10, 11). OLIG2+ progenitors from the glial lineage initiate tumors during the carcinogenesis and relapse of medulloblastoma, indicating that oncogenic networks driven by OLIG2 might be therapeutic targets (12). However, the current mortality rate from MED remains high. In order to find valid diagnostic and treatment techniques, it is critical to figure out the precise molecular pathways behind MED occurrence, growth, and recurrence.

During the previous few decades, the development of microarray technology has triggered a molecular revolution in the field of biological science. With advanced high-throughput gene sequencing technology, we have the opportunity to re-understand the genesis and development of medulloblastoma from the whole genome level. Through these new technologies, more and more molecular mechanisms and effective biomarkers are being discovered. To find possible MED biomarkers, we ran a series of analysis using high-throughput sequencing data obtained from three different datasets GSE39182, GSE74195, and GSE86574 in Gene Expression Omnibus (GEO). We first identified common DEGs from three databases and performed gene Ontology (GO), Kyoto Encyclopedia of Genes and Genomes (KEGG) pathway enrichment and protein-protein interaction (PPI) network analyses to better comprehend the molecular underpinnings of cancer initiation and development. We confirmed that AURKA and KIF20A were associated with patient prognosis and could be used as biomarkers for MED. Then, immunohistochemistry was used to confirm its presence in patients. As for the screening of Hub genes in MED, compared with previous studies, we found two new Hub genes for the first time, and identified new biomarkers AURKA and KIF20A that are critical to the prognosis of patients combined with survival data. At the same time, we used experimental methods for the first time to verify that the expression of these two proteins in human medulloblastoma tissues is indeed significantly different from that in normal brain tissues.

In conclusion, our research identifies new possible prognostic indicators and therapeutic targets for MED.

## Materials and Methods

### Immunohistochemistry

The tumor tissue of 10 medulloblastoma patients and part of cerebellar tissue of 4 cerebellar hemorrhage patients were paraformaldehyde fixed with a concentration of 4% for 24 hours before paraffin embedment. These sections were antigen-repaired with sodium citrate buffer and then sealed with goat serum. AURKA mouse monoclonal antibody (1:400, Proteintech, 66757-1-IG) and KIF20A rabbit polyclonal antibody (1:400, Proteintech, 15910-1-AP) were stained overnight at 4°C, and the second antibody was subjected to incubation for 1 hour at ambient temperature. The percentages of AURKA and KIF20A positive cells were calculated.

### Microarray Data

As a publicly accessible functional genomics resource, GEO (https://www.ncbi.nlm.nih.gov/geo/) includes high-throughput gene expression data, chips, as well as microarrays (13, 14). We downloaded three gene expression datasets (GSE39182 (15), GSE74195 (16), GSE86574 (17)) from GEO (Illumina GPL6947, Agilent GPL6480, Affymetrix GPL570 platform). Founded on the platform-based annotation information, we transformed the probes into the matching gene symbol. The GSE74195 dataset was composed of 27 MED tissue samples and 5 noncancer samples. GSE86574 contained 16 MED tissue samples and 5 noncancer samples. GSE39182 was composed of 20 MED tissue samples and 5 noncancer samples. The above datasets altogether contain 63 MED tissue samples and 15 noncancer samples.

### Identification of DEGs

GEO2R (http://www.ncbi.nlm.nih.gov/geo/geo2r) was utilized to filter the DEGs between MED and noncancer samples. GEO2R is an interactive online application for determining DEGs across test settings by comparing two or more datasets in one series of GEO. P < 0.05 and logFC > 1 or < –1 were taken as cut-off standards.

### GO and KEGG Enrichment Analyses of DEGs

GO is a key bioinformatics tool, which can be used to annotate genes and analyze the corresponding biological processes. In contrast, KEGG is a huge database for studying high-level functions and biological systems derived from massive molecular datasets collected by high-throughput test techniques. We applied Metascape (http://metascape.org/gp/index.html#/main/step1) to conduct GO and KEGG analyses on DEGs to determine their function. Metascape is an analysis tool based on web, containing discovery and annotation features (18). Moreover, the data were analyzed using online tools from the DAVID website (https://david.ncifcrf.gov/home.jsp) to guarantee the results’ legitimacy (19). The DAVID is an online bioinformatics database that includes a comprehensive biological knowledge base and analytical tools, as well as a large amount of annotation information concerning functions for genes and proteins, from which biological information may be extracted. P < 0.05 was taken as the cut-off standard.

### PPI Network Construction and Screening of Hub Genes

The PPI network of DEGs or one single gene was established by utilizing STRING online database (http://string-db.org) (20), which can be adopted to analyze the functional protein–protein connections, and can explore the processes of disease formation or progression. Cytoscape is a free bioinformatics visualization software platform for visualizing interaction networks between molecules and finding hub genes (21). The Cytoscape plug-in cytoHubba is an APP for clustering a specific topology-based network based in order to locate highly linked sections (22). We identified the hub genes by three algorithms: DEGREE, MCC, and MNC in cytoHubba.

### Target Gene Selection by Associating the Hub Gene Expression With the Survival of Patients with MED

To study the relationship between the hub gene expression and MED patients’ prognosis, we screened two datasets (GSE30074 and GSE85217) containing patient survival information and gene expression information from GEO database. Then, we used GraphPad Prime software to conduct a survival analysis on the hub genes.

## Results

### Identification of DEGs in MED

**Figure 1:**
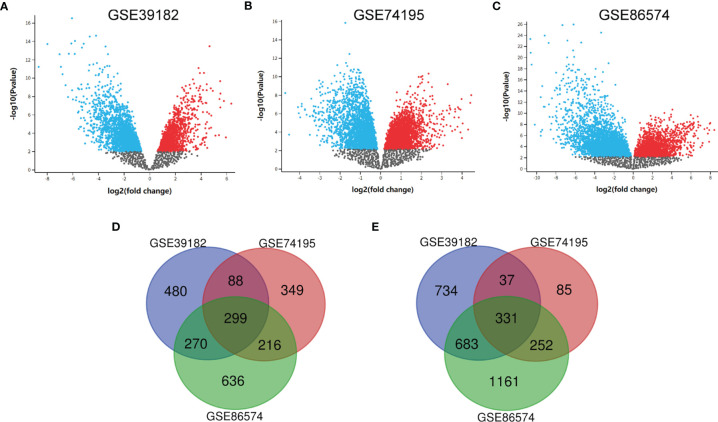
Identification of DEGs shared between the three databases. **(A)** The volcano map of GSE39182, **(B)** The volcano map of GSE74195, **(C)** The volcano map of GSE86574, **(D)** A Venn diagram used to identify 299 upregulated target genes in MED. **(E)** A Venn diagram used to identify 331 downregulated target genes in MED.

After the microarray results were standardized, DEGs (3470 in GSE39182, 1657 in GSE74195 and 3848 in GSE86574) were determined by GEO2R. The data were filtered by logFC ≥1 or ≤ –1 and P < 0.05. We analyzed the DEGs in each dataset separately and showed them in volcano map (**Figures 1A–C**). As indicated in the Venn diagram, the overlap between the three datasets includes 630 genes, with 299 upregulated genes (**Figure 1D**) and 331 downregulated genes (**Figure 1E**) between MED tissues and noncancer tissues.

### GO and KEGG Enrichment Analyses of DEGs

**Figure 2:**
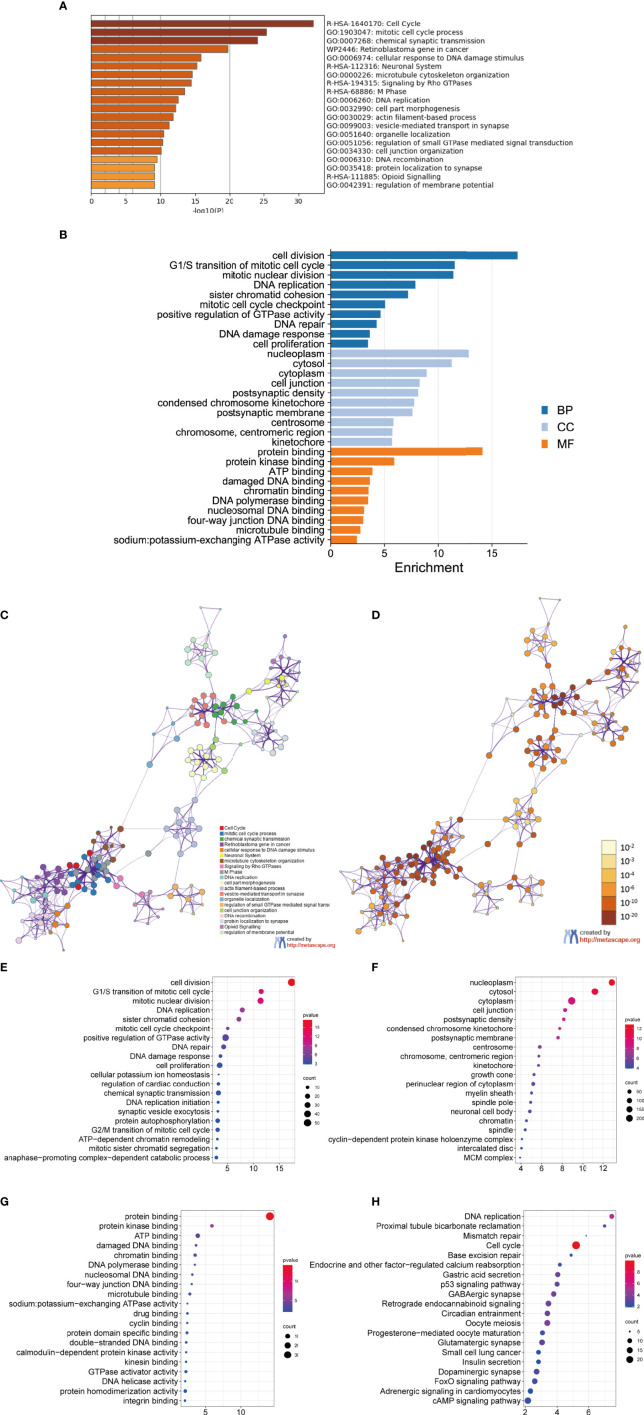
GO analysis and KEGG pathway analysis of DEGs. **(A–D)** GO analysis and KEGG pathway analysis. Bubble diagram of BP **(E)**, CC **(F)**, MF **(G)** and KEGG **(H)** analysis for MED.

To better understand DEGs’ biological classification, Metascape online tools (http://metascape.org) were adopted for GO and KEGG pathway enrichment analyses. It is found that DEGs were predominantly enriched in the cell cycle, mitotic cell cycle process, chemical synaptic transmission, retinoblastoma gene in cancer, and neuronal system (**Figures 2A–D**). Afterwards, we conducted GO analysis by using the DAVID website. The biological process changes of DEGs were significantly concentrated in cell division, G1/S transition of mitotic cell cycle, mitotic nuclear, DNA replication, and sister chromatid cohesion (**Figure 2E**). The cell component (CC) changes of DEGs were mostly concentrated in nucleoplasm, cytosol, cytoplasm, cell junction, and postsynaptic density (**Figure 2F**). The molecular function (MF) changes of DEGs were mostly concentrated in protein biding, protein kinase binding, ATP binding, damaged DNA binding, as well as chromatin binding (**Figure 2G**). We investigated the DEG-enriched KEGG pathways further using the DAVID website. We discovered that the DEG genes were mostly concentrated in the cell cycle, DNA replication, oocyte meiosis, GABAergic synapse, and retrograde endocannabinoid signaling (**Figure 2H**).

### PPI Network Construction and Screening of Hub Genes and Analysis

**Figure 3:**
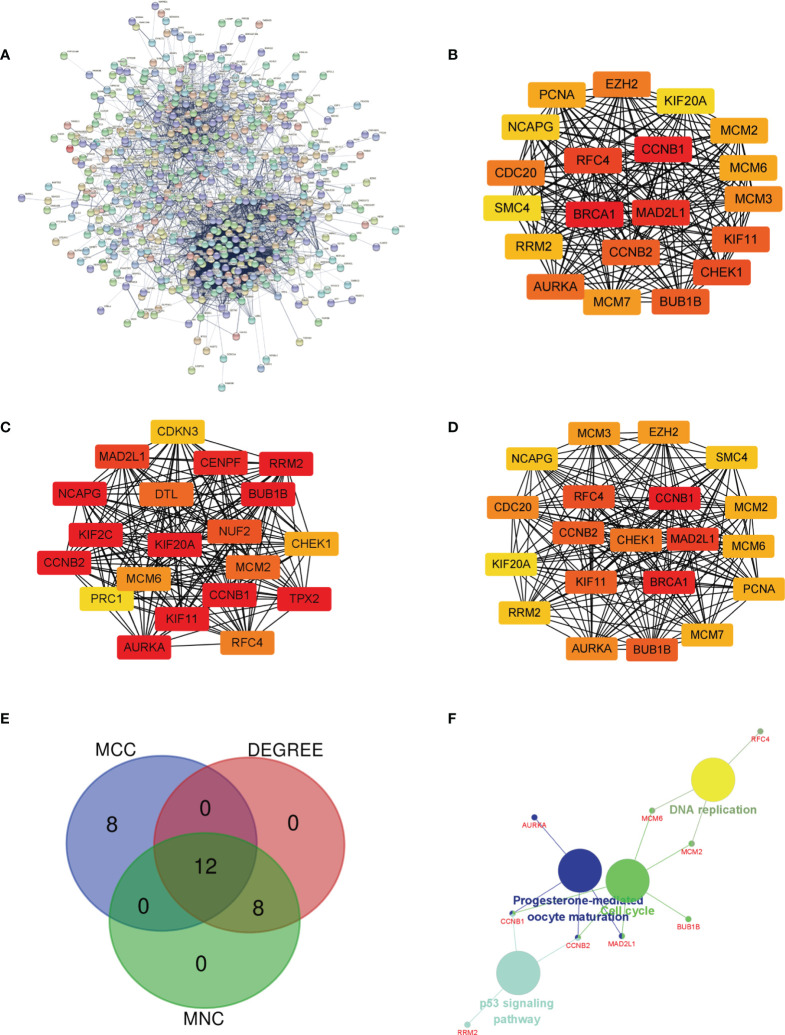
Determination of the hub genes. **(A)** PPI network of DEGs in MED. **(B–D)** Three different metrics: DEGREE, MCC, and MNC. **(E)** In MED, 12 hub genes were identified using a Venn diagram, **(F)** The ClueGO plugin in Cytoscape software was used to visualize the findings of KEGG pathway analysis.

The PPI network of DEGs was built (**Figure 3A**), and the most important module was determined by the STRING online tool. Then, based on the DEGREE (**Figure 3B**), MCC (**Figure 3C**), and MNC (**Figure 3D**) in cytoHubba, we identified 12 hub genes. They were AURKA, BUB1B, CCNB1,CCNB2, CHEK1, KIF11, KIF20A, MAD2L1, MCM6, NCAPG, RFC4, and RRM2 (**Figure 3E** venn). The full names, abbreviations, as well as their functions for these hub genes are tabulated in **Table 1**. To visualize the findings of KEGG path analysis, we utilized the ClueGO plugin for Cytoscape. (**Figure 3F**).

**Table 1:** Functional roles of 12 hub genes.

NO.	Gene symbol	Full name	Function
1	KIF20A	kinesin family member 20A	Mitotic kinesin is necessary for cytokinesis mediated by the chromosomal passenger complex (CPC).
2	AURKA	aurora kinase A	A cell cycle-regulated kinase that appears to be involved in the production and/or stability of microtubules at the spindle pole during chromosomal segregation.
3	BUB1B	threonine kinase B	Essential component of the mitotic checkpoint
4	RRM2	ribonucleotide reductase M2	Provides the precursors necessary for DNA synthesis.
5	KIF11	kinesin family member 11	During mitosis, a motor protein is essential for the formation of a bipolar spindle.
6	MAD2L1	MAD2 mitotic arrest deficient-like 1	A component of the spindle-assembly checkpoint that prevents anaphase from occurring until all chromosomes are correctly aligned at the metaphase plate.
7	CCNB1	cyclin B1	Belongs to the cyclin family and is required for cell cycle regulation during the G2/M (mitosis) transition.
8	RFC4	replication factor C4	Involved in the elongation of the multiprimed DNA template
9	NCAPG	non-SMC condensin I complex, subunit G	Condensin complex regulatory subunit, a complex essential for the conversion of interphase chromatin into mitotic-like condensed chromosomes.
10	CCNB2	cyclin B2	Belongs to the cyclin family and is required for cell cycle regulation during the G2/M (mitosis) transition.
11	MCM6	minichromosome maintenance complex component 6	one of the highly conserved mini-chromosome maintenance proteins (MCM) required for eukaryotic genome replication to begin
12	CHEK1	checkpoint kinase 1	Serine/threonine protein kinase that is necessary for checkpoint-mediated cell cycle arrest and DNA repair activation in the presence of DNA damage or unreplicated DNA.

### Determination of AURKA and KIF20A as the Target Genes by Survival Analysis

**Figure 4:**
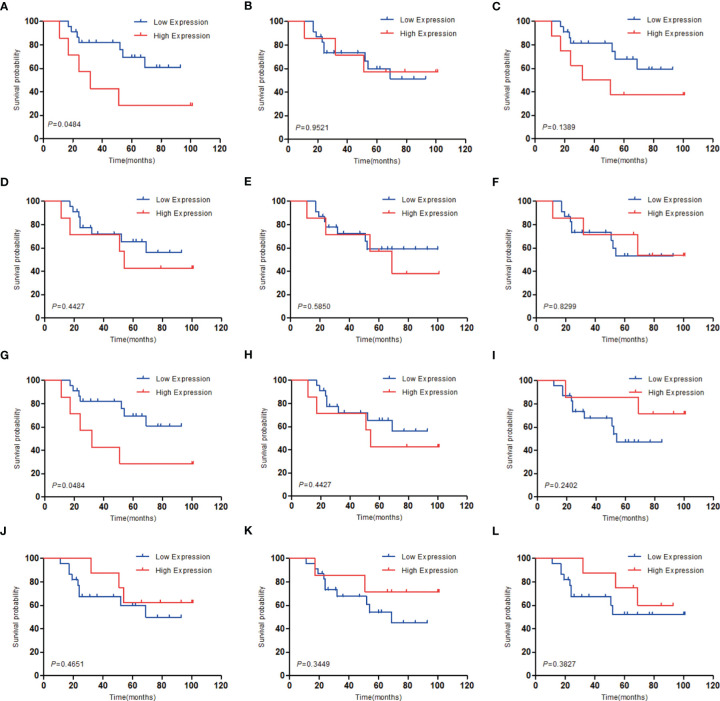
Survival analysis of the 12 hub genes in MED based on the GSE30074 database. **(A)** AURKA, **(B)** BUB1B, **(C)** CCNB1, **(D)** CCNB2, **(E)** CHEK1, **(F)** KIF11, **(G)** KIF20A, **(H)** MAD2L1, **(I)** MCM6, **(J)** NCAPG, **(K)** RFC4, **(L)** RRM2; P < 0.05 was considered statistically significant.

**Figure 5:**
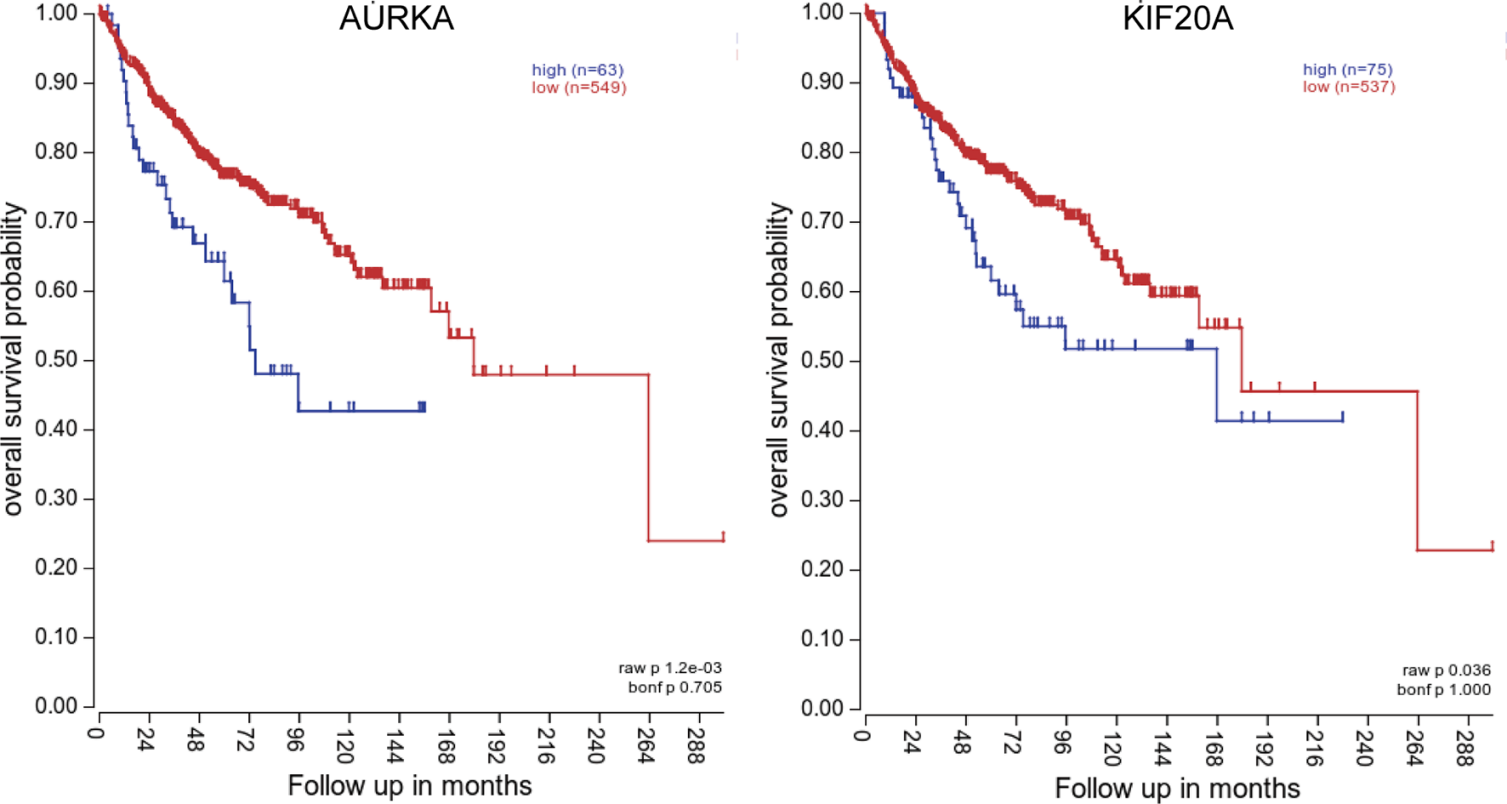
Survival analysis of AURKA and KIF20A based on the GSE85217 database. **(A)** AURKA, **(B)** KIF20A. P < 0.05 was considered statistically significant.

**Figure 6:**
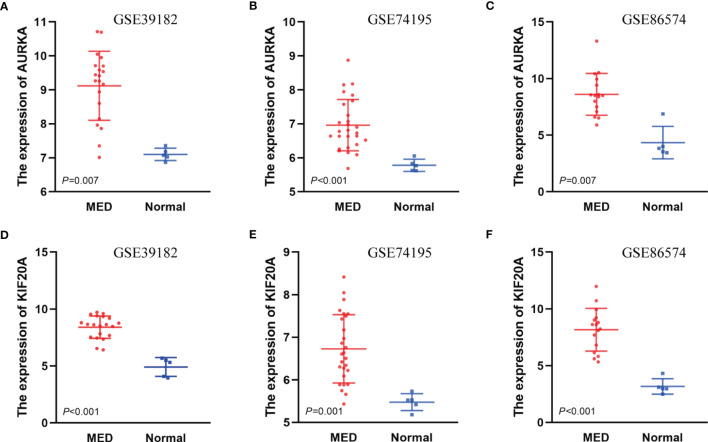
The different expressions of AURKA and KIF20A in the three databases. **(A)** Expressions of AURKA in GSE39182, **(B)** Expressions of AURKA in GSE74195, **(C)** Expressions of AURKA in GSE86574, **(D)** Expressions of KIF20A in GSE39182, **(E)** Expressions of KIF20A in GSE74195, **(F)** Expressions of KIF20A in GSE86574.

To investigate the relationship between hub genes and MED patient’s survival, we conducted a survival analysis of the hub genes by using survival data from GSE30074 (**Figures 4A–L**). We discovered that overexpressions of AURKA and KIF20A expression were linked with survival in MED patients. We then used the survival data of GSE85217 to verify these two genes again (**Figure 5**). Therefore, AURKA and KIF20A were selected as target genes. The different expressions of AURKA and KIF20A in the three databases were shown (**Figures 6A–F**).

### The Biological Role of AURKA in Tumors

**Figure 7:**
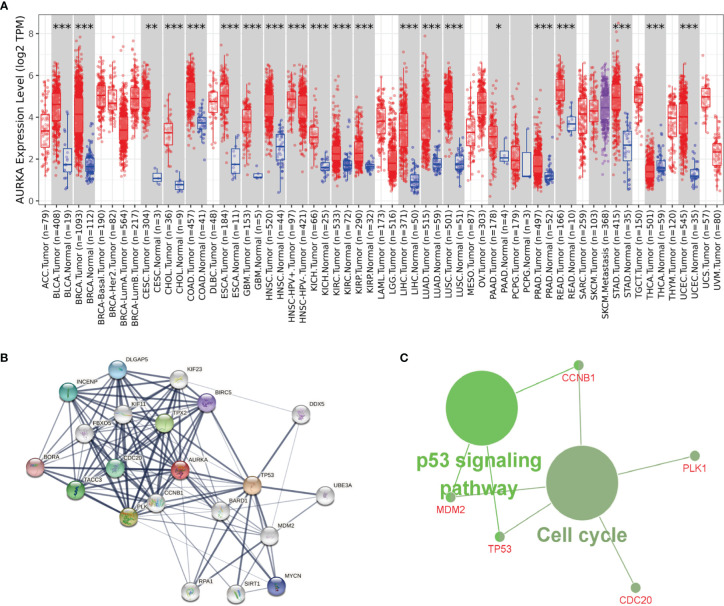
AURKA’s biological involvement in tumors. **(A)** AURKA expression in different tumors. **(B)** AURKA gene STRING interaction network preview of interacting proteins (showing top 20 STRING interactants). **(C)** Illustration of KEGG pathway analysis findings using the ClueGO plugin in Cytoscape. *P < 0.05, **P < 0.01, ***P < 0.001.

To study whether AURKA is used as a carcinogene in other tumors, we investigated the differential expression of AURKA in several tumors and healthy tissues using GEPIA (http://gepia.cancer-pku.cn/). We found that AURKA is upregulated in many tumors, such as BLCA and CHOL (**Figure 7A**). To investigate the underlying molecular processes of AURKA, we utilized the STRING website to identify genes owning a protein–protein interaction with AURKA (**Figure 7B**). Using Metascape online tools, we obtained the enrichment analysis results of AURKA and the top 20 interacting proteins pathways and biological processes (**Table 2**), and then we employed the ClueGO plugin for Cytoscape to visualize the findings of KEGG path analysis of these linked genes. It is discovered that AURKA-related genes are mostly concentrated in the cell cycle and p53 signaling pathway (**Figure 7C**). We discovered that AURKA has a significant coexpression relationship with CCNB1, PLK1, CDC20, TP53, and MDM2.

**Table 2:** Pathway and process enrichment analysis of AURKA and top 20 interacting proteins.

GO	Category	Description	Count	-Log10(P)
M129	Canonical Pathways	PID PLK1 PATHWAY	12	29.03
M14	Canonical Pathways	PID AURORA B PATHWAY	11	26.91
GO:0051301	GO Biological Processes	cell division	17	26.77
R-HSA-69278	Reactome Gene Sets	Cell Cycle, Mitotic	17	25.8
GO:0051321	GO Biological Processes	meiotic cell cycle	8	11.64
GO:0061640	GO Biological Processes	cytoskeleton-dependent cytokinesis	6	10.37
hsa04914	KEGG Pathway	Progesterone-mediated oocyte maturation	6	10.18
CORUM:6184	CORUM	AuroraB-AuroraC-INCENP complex	3	9.54
GO:0051656	GO Biological Processes	establishment of organelle localization	7	8.64
GO:0051988	GO Biological Processes	regulation of attachment of spindle microtubules to kinetochore	3	7.08
GO:0001578	GO Biological Processes	microtubule bundle formation	3	4.13

### The Biological Role of KIF20A in Tumors

**Figure 8:**
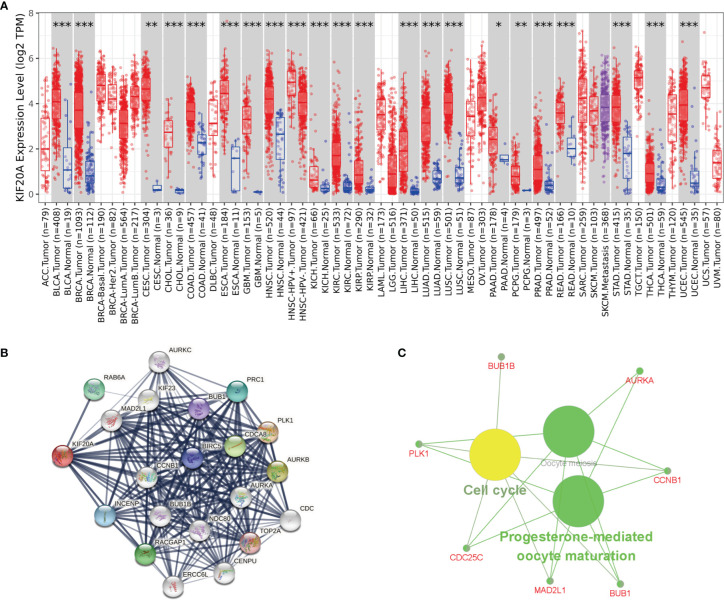
KIF20A’s biological involvement in tumors. **(A)** KIF20A expression in different tumors. **(B)** KIF20A gene STRING interaction network preview of interacting proteins (showing top 20 STRING interactants). **(C)** Illustration of KEGG pathway analysis findings using the ClueGO plugin in Cytoscape. *P < 0.05, **P < 0.01, ***P < 0.001.

To study whether KIF20A is used as a carcinogene in other tumors, we analyzed the differential expression of KIF20A in several tumors and healthy tissues using GEPIA. We found that the KIF20A overexpression occurs in many tumors, such as BLCA and COAD (**Figure 8A**). To investigate the underlying molecular processes of KIF20A, we utilized the STRING website to identify genes owning a protein–protein interaction with KIF20A (**Figure 8B**). Using Metascape online tools, we obtained the enrichment analysis results of KIF20A and the top 20 interacting proteins pathways and biological processes (**Table 3**), and then we employed the ClueGO plugin for Cytoscape to visualize the findings of KEGG path analysis of these linked genes. It is discovered that KIF20A-related genes are mostly concentrated in the cell cycle, oocyte meiosis, and progesterone-mediated oocytematuration (**Figure 8C**). We discovered that KIF20A has a significant coexpression relationship with BUB1, BUB1B, CCNB1, MAD2L1, PLK1, AURKA and CDC25C.

**Table 3:** Pathway and biological process enrichment analysis of KIF20A and top 20 interacting proteins.

GO	Category	Description	Count	-Log10(P)
GO:0007051	GO Biological Processes	spindle organization	11	19.88
R-HSA-1640170	Reactome Gene Sets	Cell Cycle	14	18.02
M129	Canonical Pathways	PID PLK1 PATHWAY	8	17.52
M242	Canonical Pathways	PID AURORA A PATHWAY	7	16.18
R-HSA-69620	Reactome Gene Sets	Cell Cycle Checkpoints	9	12.75
R-HSA-3108232	Reactome Gene Sets	SUMO E3 ligases SUMOylate target proteins	7	10.56
R-HSA-6804756	Reactome Gene Sets	Regulation of TP53 Activity through Phosphorylation	6	10.45
WP1984	WikiPathways	Integrated breast cancer pathway	6	9.07
GO:0060236	GO Biological Processes	regulation of mitotic spindle organization	4	7.86
GO:0030162	GO Biological Processes	regulation of proteolysis	8	7.7
GO:0090307	GO Biological Processes	mitotic spindle assembly	4	7.52
GO:1903800	GO Biological Processes	positive regulation of production of miRNAs involved in gene silencing by miRNA	3	7.2
GO:0051347	GO Biological Processes	positive regulation of transferase activity	7	7.18
GO:0010639	GO Biological Processes	negative regulation of organelle organization	4	4.12

### The Expression level of AURKA and KIF20A in MED

**Figure 9:**
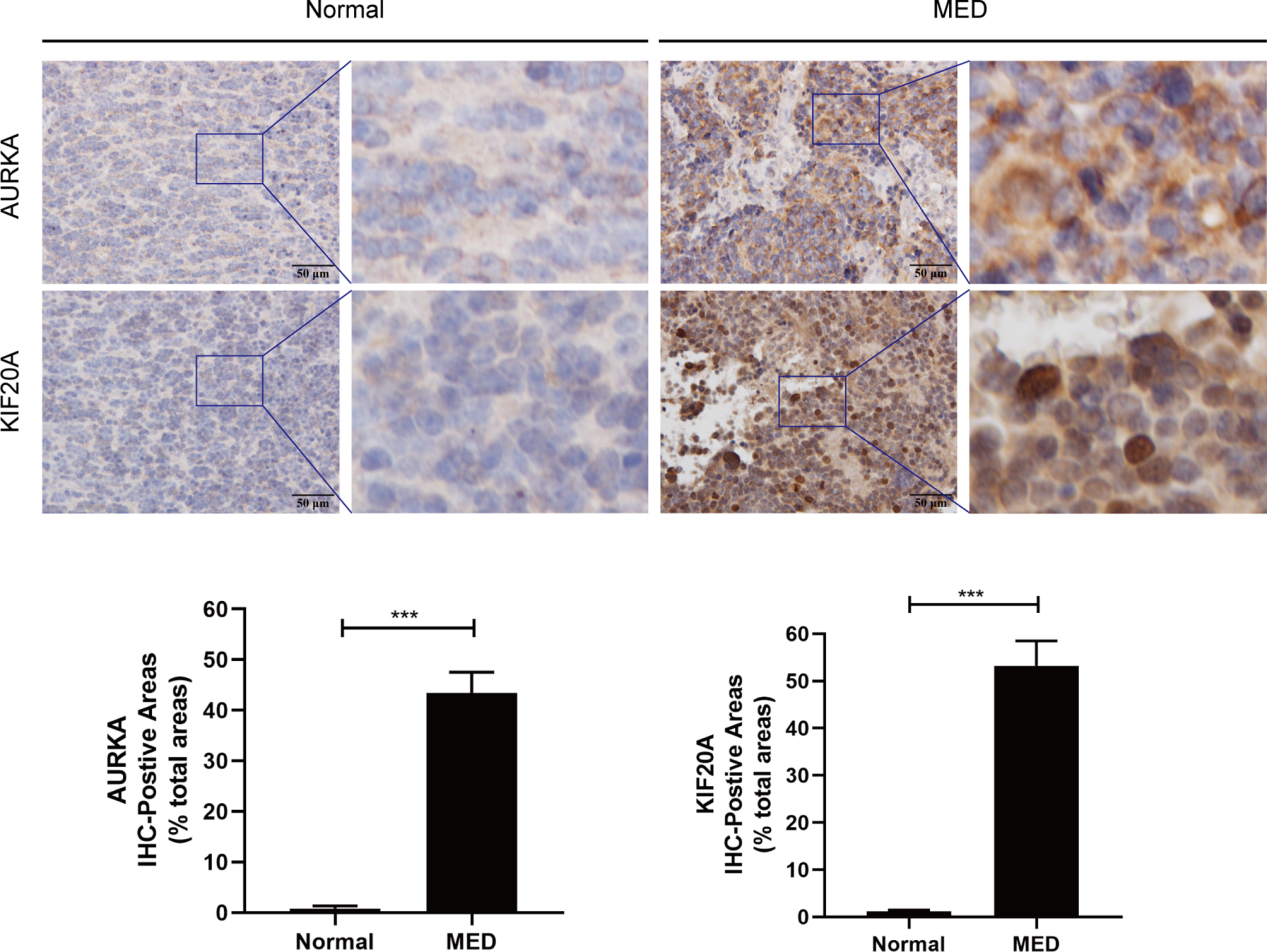
Immunohistochemistry analysis of AURKA and KIF20A expression in Medulloblastoma tissues from Tianjin Huanhu Hospital (normal brain, n = 4; MED, n = 10). Scale bar = 50μm. Below is a list of statistical quantitative analyses. Data are mean ± SD. ***P < 0.001, one-way ANOVA.

To verify the expression of AURKA and KIF20A at the protein level, IHC staining was carried out, and it was discovered that AURKA and KIF20A expression levels in MED were substantially higher than in cerebellar tissue (**Figure 9**). AURKA and KIF20A were strongly positive in MED group, while in normal brain tissue, they were both expressed as weakly positive or negative.

## Discussion

Brain tumors cause the most fatalities related to cancer in children, and the most prevalent malignant juvenile brain tumor is medulloblastoma (MB). Medulloblastomas account for 10% of all childhood brain tumors. These tumors occur only in the posterior fossa and have the possibility of mild meningeal spread (23). Surgical surgery, adjuvant chemotherapy, and craniospinal irradiation are the current therapeutic options (24). In spite of these advancements, 25–30% of patients still die from the condition, and those who survive have substantial long-range negative effects induced by intensive therapy (8), such as severe motor and cognitive deficits. As a result, it is urgent to discover new biomarkers and design new therapy procedures based on the presence of specific hub genes. Progresses in high-throughput chip technology and bioinformatics methods have helped us identify hub genes and provide a deeper understanding of childhood medulloblastoma.

In this study, DEGs between medulloblastoma tissue and healthy brain tissue were discovered analyzing three mRNA microarray datasets. The three datasets yielded 630 DEGs, comprising 299 upregulated genes and 331 downregulated genes. First, we performed GO (BP, CC and MF) analysis on 630 DEGs to study their biological functions. DEGs were mostly enriched in the cycle process, mitotic cell, cell cycle, chemical synaptic transmission, retinoblastoma gene in cancer, and neuronal system. Specifically, the changes in biological processes (BP) of DEGs were significantly enriched in Cell division, DNA replication, mitotic nuclear, G1/S transition of mitotic cell cycle, and sister chromatid cohesion. CC changes of DEGs were largely enriched in nucleoplasm, cytosol, cytoplasm, cell junction, and postsynaptic density. MF changes were largely enriched in protein binding, protein kinase binding, ATP binding, damaged DNA binding, and chromatin binding. These findings imply that these genes function in medulloblastoma cell mitosis, invasion, and metastasis. Based on KEGG pathway analysis results, DEGs were shown to be largely enriched in cell cycle, DNA replication, oocyte meiosis, GABAergic synapse and retrograde endocannabinoid signaling. Understanding the fundamental processes of MED proliferation and invasion, as well as predicting the course of MED, will be aided by research into these pathways.

We constructed a PPI network with these differential genes, and then screened out 12 hub genes: AURKA, BUB1B, CCNB1,CCNB2, CHEK1, KIF11, KIF20A, MAD2L1, MCM6, NCAPG, RFC4, and RRM2. KEGG analysis of these hub genes illustrated that they were largely enriched in DNA replication, cell cycle, progesterone-mediated oocyte maturation, and P53 signaling pathway. These hub genes can be potentially utilized as therapeutic targets for medulloblastoma. Then, based on the prognostic information of medulloblastoma patients in the GSE30074 and GSE85217 datasets. The expression levels of AURKA and KIF20A were discovered to be linked with the prognosis of medulloblastoma patients. Therefore, AURKA and KIF20A were selected as the target genes in this research.

To analyze the expression of AURKA and KIF20A in medulloblastoma at the protein level, we selected MED tissue samples and normal cerebellum tissues for immunohistochemical staining, and the findings proved that the expression of AURKA and KIF20A in cerebellum tissues was weakly positive or negative, while in MED, both AURKA and KIF20A were overexpressed, which was the same as their mRNA expression in MED. It was proved that AURKA and KIF20A can be used as biomarkers for MED.

KIF20A, also known as mitotic kinesin-like protein 2 (MKlp2) or rabkinesin6 (RAB6KIFL), is a kinesin-6 family 10 microtubule plus-end directed motor that is also necessary in the exit of mitosis for the final step of cytokinesis (25). KIF20A is found in the central spindle during mitosis, whose phosphorylation is necessary for cytoplasmic division (26). It has recently been reported that KIF20A knockout results in loss of neural precursor cells and neurons during cortical neurogenesis owing to premature cell cycle exit and neuronal differentiation (27). The overexpression of KIF20A is linked to the onset, progression, and prognosis of different cancers. Recent researches have reported that KIF20A was upregulated in esophageal squamous cell carcinoma (28), lung adenocarcinoma (29), prostate cancer (30), cervical cancer (31), colorectal cancer (32), non-small cell lung cancer (33), gastric cancer (34), bladder cancer (35), renal clear cell carcinoma (36), breast cancer (37), hepatocellular carcinoma (38), nasopharyngeal carcinoma (39), ovarian cancer (40), leukemia (25), glioma (41), and soft tissue sarcoma (42). This is consistent with our analysis in the GEPIA online database. KIF20A overexpression is linked to HCC and NPC patient survival, and it can be utilized independently as a prognostic marker for HCC and NPC patients (38, 39). Furthermore, KIF20A is linked with chemotherapy resistance and radiation resistance of some tumors. For instance, in colorectal cancer, KIF20 is highly upregulated in oxaliplatin resistant cell lines, and has a close correlation with the survival of colorectal cancer patients. Silencing KIF20A increased the sensitivity of cells to oxaliplatin *in vivo* and *in vitro* (43). Forkhead box M1 (FOXM1), a transcription factor participating in cell proliferation as well as cycle progression, has been linked to chemotherapy sensitivity. High expression of FOXM1 may increase docetaxel resistance by promoting KIF20A expression (44). FOXM1 plays a role in regulating radiosensitivity in glioma and breast cancer cells, and FOXM1 may enhance radiation resistance in part by inducing KIF20A expression (45). In addition to the high expression of KIF20A in the above tumors, our study found that KIF20A was also highly upregulated in MED, and according to the prognostic survival analysis, KIF20A was shown to be directly linked to the prognosis of MED patients, and the overexpression of KIF20A illustrated a poor survival prognosis of MED patients. KEGG enrichment analysis of KIF20A revealed that KIF20A and its associated genes were shown to be highly enriched in the cell cycle, oocyte meiosis, and progesterone-mediated oocytematuration. The enrichment analysis results of KIF20A and the top 20 interacting proteins pathways and biological processes were shown in **Table 3**. Further study on these pathways will assist to reveal the mechanism of KIF20A in MED.

AURKA is a serine/threonine kinase whose activation is required for cell division through controlling mitosis. Under physiological conditions, AURKA controls cilia breakdown, neurite extension, cell movement, DNA replication, and aging procedures. It is found in mitochondria in a cancer-like environment, where mitochondrial dynamics and ATP generation are affected, actively promoting DNA repair and cell migration and invasion (46). Our analysis using the GEPIA database showed that AURKA was highly upregulated in various tumors. The function of AURKA substrates, some of which are mitotic regulators, tumor suppressors, or carcinogenes, is modulated by AURKA-mediated phosphorylation (47). Currently many researches have revealed that the activation of AURKA has a crucial role in multiple cancers, for example, gastric cancer (48), liposarcoma (49), neuroblastoma (50), pancreatic cancer (51), gastrointestinal cancer (52), hepatocellular carcinoma (53), leukemia (54), epithelial ovarian cancer (55), head and neck squamous cell carcinoma (56), prostate cancer (57), bladder cancer (58), upper gastrointestinal adenocarcinoma (59), fertile tumor of the bone marrow (60), oral squamous cell carcinoma (61), KIF20A was highly expressed. In most tumors, upregulation of KIF20A suggests a poor prognosis of patients, except for colon cancer, where it has been found that the AURKA upregulation in colon cancer suggests better prognosis of patients with colon cancer (62). AURKA overexpression contributed to oxaliplatin-induced death of colon cancer cells, while AURKA knockdown drastically reduced chemotherapy sensitivity of colon cancer cells to oxaliplatin. In terms of mechanism, AURKA inhibits DNA damage responses in a TP53-dependent manner by inhibiting the expression of multiple DNA damage repair genes, which may partly explain ARUKA’s association with beneficial outcomes in colon cancer. In addition to the upregulation of AURKA in the above tumors, our study found that AURKA was also overexpressed in MED. According to the prognostic survival analysis, AURKA was closely linked to the prognosis of MED patients, and the AURKA upregulation predicted a poor survival prognosis of MED patients. By KEGG enrichment analysis of AURKA and its interacting proteins, we found that these proteins affect typical carcinogenic pathways like the cell cycle and p53 signaling pathway. To better understand the biological functions of AURKA, enrichment analysis was performed using Metascape online tools, the enrichment analysis results of AURKA and the top 20 interacting proteins pathways and biological processes were shown in **Table 2**. All of these data points to AURKA as a potential target for cancer treatment, and various small compounds targeting AURKA have been found. These AURKA inhibitors (AKIs) have been studied in preclinical investigations, and some have been studied in clinical trials as monotherapies or in conjunction with traditional chemotherapy or other targeted medicines.

According to the latest research, we discussed the involvement of KIF20A and AURKA in the occurrence and development of MED and their close correlation with the prognosis of patients, implying that these genes might be used as promising biomarkers and therapeutic targets of MED. Despite the fact that our work adds to our understanding of the link between AURKA, KIF20A and MED, it has several limitations. First of all, the mRNA sequencing data of MED in this study only came from GEO database. As there were few sequencing data related to MED, our sample size was insufficient. Secondly, the basic experiments of our verification and inspection are insufficient. Only IHC experiments are involved, and RT-qPCR validation in clinical samples is also required. *In vitro* and *in vivo* experiments are also our next research direction. Third, for these genes play a role in the mechanism is still not entirely clear, Our current study only stays at the transcriptional level and does not involve the upstream and downstream pathways of target gene related proteins, the next step we should take multiple omics research, including proteomics, metabolomics, and DNA methylation, autophagy and LncRNA, ceRNA, etc. Fourth, Immune infiltration is important in the growth of tumors. the immune microenvironment profoundly affected the prognosis of patients with tumor, because our data is limited, the present study did not show that the target gene influences the immune cell migration and invasion, the next step should be studied gradually AURKA, KIF20A biological function in immune microenvironment. Fifthly, our study is limited to MED. Next, we can carry out combinative analysis with a variety of tumors to explore the mechanism of action of AURKA and KIF20A in generalized cancer. Sixthly, to explore the correlation between target genes and patients’ prognosis and survival, we need to consider multiple clinical factors and parameters, such as the details of patients’ treatment. However, such information is lacking in the public database. We only included the information of two datasets, and the sample size is insufficient, which may lead to analysis bias, which is also the biggest problem of this study. In the next step, we should conduct a prospective study to avoid insufficient sample size due to the retrospective nature. At the same time, as many environmental factors and genetic factors including region, race, age, gender, family history were included as much as possible.

## Conclusions

In conclusion, this study found that DEGs participate in the occurrence and development of MED through a public database study, and screened out two possible biomarkers, AURKA and KIF20A. Both genes may be new therapeutic targets. In addition, basic experiments on clinical patient specimens proved that these two genes were indeed upregulated in MED tissues. However, the biological function of these genes in MED remains to be further studied.

## Data Availability Statement

The original contributions presented in the study are included in the article/supplementary material. Further inquiries can be directed to the corresponding authors.

## Ethics Statement

The studies involving human participants were reviewed and approved by Tianjin Huanhu Hospital Ethics Committee (Tianjin, China). Written informed consent to participate in this study was provided by the participants’ legal guardian/next of kin. Written informed consent was obtained from the individual(s), and minor(s)’ legal guardian/next of kin, for the publication of any potentially identifiable images or data included in this article.

## Author Contributions

BL, HY, and XT: study design. BL, YZ, and JJ: data collection. BL, LX, and QW: data analysis and interpretation. BL, YZ, and JJ: writing, review, polishing, and revision of the manuscript. All authors contributed to the article and approved the submitted version.

## Funding

This study was financially supported by grants from the National Natural Science Foundation of China (No. 81972349), Tianjin Municipal Science and Technology Commission (No. 20JCQNJC00410), Tianjin Health Science and Technology Project (TJWJ2021MS030), and Applied basic research project of Tianjin Science and Technology Bureau (21JCZDJC00460).

## Conflict of Interest

The authors declare that the research was conducted in the absence of any commercial or financial relationships that could be construed as a potential conflict of interest.

## Publisher’s Note

All claims expressed in this article are solely those of the authors and do not necessarily represent those of their affiliated organizations, or those of the publisher, the editors and the reviewers. Any product that may be evaluated in this article, or claim that may be made by its manufacturer, is not guaranteed or endorsed by the publisher.
